# Clinical learning environment measurement for medical trainees at transitions: relations with socio-cultural factors and mental distress

**DOI:** 10.1186/1472-6920-14-226

**Published:** 2014-10-21

**Authors:** Jer-Chia Tsai, Cheng-Sheng Chen, I-Feng Sun, Keh-Min Liu, Chung-Sheng Lai

**Affiliations:** Department of Internal Medicine, Kaohsiung Medical University Hospital, Faculty of Renal Care, College of Medicine, Kaohsiung Medical University, Kaohsiung, Taiwan; Department of Psychiatry, Kaohsiung Medical University Hospital, School of Medicine, College of Medicine, Kaohsiung Medical University, Kaohsiung, Taiwan; Department of Surgery, Kaohsiung Municipal Hsiao-Kang Hospital, Kaohsiung Medical University, Kaohsiung, Taiwan; Department of Anatomy, School of Medicine, College of Medicine, Kaohsiung Medical University, Kaohsiung, Taiwan; Department of Surgery, Kaohsiung Medical University Hospital, School of Medicine, College of Medicine, Kaohsiung Medical University, No. 100, Tz-You First Road, San-Ming District, Kaohsiung, 80708 Taiwan

**Keywords:** Clinical learning environment, Medical trainees, Socio-cultural factors, Medical training settings, Mental distress

## Abstract

**Background:**

Measuring clinical learning environment is crucial for the quality improvement of medical education, especially for medical trainees at transition stages. Medical education in Taiwan is shaped by multiple socio-cultural influences. The aims of this study were to construct an instrument for measuring clinical learning environment in alignment with the local socio-cultural factors and medical training settings, and further investigate the relationship between the quality of the clinical learning environment and the status of mental distress among medical trainees.

**Methods:**

Participants consisted of 189 medical trainees (62 interns, 52 postgraduate year one (PGY1) residents, and 75 senior residents). Instruments included a designed 40-item Clinical Learning Environment Questionnaire (CLENQ) and a five-item Brief Symptoms Rating Scale (BSRS-5) for evaluating mental distress. Constructs of CLENQ were examined using factor analysis. Correlations were calculated between BSRS-5 and CLENQ across the three levels of medical trainees.

**Results:**

Factor analysis of CLENQ yielded five factors- I: Teaching (13 items), II: Workload (7 items), III: Relationship pressure (9 items), IV: Organisational support (4 items) and V: Mutual trust (6 items). Intern trainees reported the lower total CLENQ scores in comparison to PGY1 and senior resident trainees. Mental distress using BSRS-5 was negatively correlated with total CLENQ scores and several key factors in all three groups.

**Conclusions:**

Our study using CLENQ has identified five major factors of clinical learning environment that are closely linked with our local socio-cultural factors and medical training settings. Medical trainee’s mental distress status was negatively correlated with the quality of CLENQ. These findings have socio-cultural relevance and medical contextual significance and might be applicable to other countries. It warrants further study to investigate the impact of clinical learning environment improvement on the medical trainee’s mental distress and performance.

## Background

Measuring an educational or learning environment has become an essential approach for evaluating the quality of the medical curriculum and training programmes by identifying the strengths, weaknesses, and priority areas for improvement [1–3]. A systemic review has reported the validity and usefulness of several instruments to measure the clinical educational environment [3]. Among them, the Postgraduate Hospital Educational Environment Measure (PHEEM) is a widely used instrument that consists of three subscales of perceptions of role autonomy, teaching, and social support [4]. Its validity and reliability have been well established at different levels of medical trainees in the various settings [3, 5, 6]. Further studies from different countries have demonstrated different dimensions and distinct interpretations of each subscale compared to the original report [4, 6–8]. Recently developed instruments have further identified more subscales for measuring clinical learning climates. For example, the Dutch Residency Educational Climate Test yielded the additional factors of “supervision, coaching and assessment, feedback, and teamwork” [9]. A clinical learning evaluation questionnaire for Arabic undergraduate clinical education exhibited the significant factors of provision of clinical cases, authenticity of clinical experiences, supervision, and organisation of doctor-patient encounter [10]. The literatures illustrate how various approaches of measuring learning environment may identify the specific factors relevant to different medical and socio-cultural contexts.

Among the three components of the educational environment defined by Mohanna et al. [11], the emotional and intellectual components of the educational environment are intangible and challenging with regard to complex learning experiences in the sense of security, mutual interactions with patients and working teams within system-based medical practice [2, 11]. Schönrock-Adema et al. [12] have identified the key elements of personal development/goal direction, relationships, and systemic maintenance and system change as a theoretic framework in assessing the educational environment of medical learning.

Clinical workplace was considered as learning environment, since it encompasses dynamic interpersonal interactions and socio-cultural contextual factors [13, 14]. An international study by Hofstede [15] has indicated that a greater power distance and uncertainty avoidance were noted in Asian culture compared to Western societies, and these cultural differences may create tense interrelationships among trainees, staff and supervisors. Schein [16] also emphasized the distinct organisational culture and subcultures of the doctors in the hospital system. Notably, the Taiwan’s medical education system has been shaped under the influences of Western and Japanese medical education systems and standards in conjunction with traditional Chinese medicine and socio-cultural influences [17]. Thus, it is necessary to incorporate socio-cultural factors and local medical training settings in designing and measuring the clinical learning environment and to identify the underlying interrelationships among learners within working teams, the organisational system and the dynamic change of the clinical educational context [18–20].

Another important concern is to explore the relationship between mental distress with the perception of the clinical learning environment. Medical students and junior doctors may encounter greater challenges and difficulties in adapting to major transitions of medical education stages from non-clinical to clinical training and from medical graduates to junior and senior doctor training [21]. These challenges include intensive clinical workload, psychological stress, difficulties in communicating within multi-professional teams, and lack of adequate support and mentoring [9, 22–25]. These detrimental effects not only affect the quality of clinical learning environment but also threaten the learning effectiveness, physical and mental health of medical trainees, as well as the outcome of patient care [26–29]. A recent national survey in Taiwan has reported that the first-year postgraduate medical trainees have experienced burnout attributed to job stress and long working-hours [30]. To measure the mental distress of our learners, we apply the instrument of five-item Brief Symptom Rating Scale (BSRS-5), a valid and reliable screening tool of mental distress in the general population and healthy workers [31, 32].

Thus, the aims of this study were: (1) to construct a clinical learning environment instrument by incorporating our socio-cultural factors in alignment with the local medical training context; (2) to investigate the perceptions of clinical learning environment and mental distress among medical trainees at different levels of clinical education stages; (3) to investigate the relationship between quality of clinical learning environment and mental distress status among medical trainees.

## Methods

### Participants

This study was conducted at the Kaohsiung Medical University Hospital in Taiwan in 2011. Medical trainees consisted of 145 interns, 87 postgraduate year one (PGY1) residents, and 218 senior residents above the second year (Rs), considered to be at the transitions stages of medical education. All of them were invited to participate in this study. Altogether, 189 medical trainees (62 interns, 52 PGY1 residents, and 75 senior residents) responded and completed the anonymous questionnaires. The research protocol was approved by the Institutional Review Board of Kaohsiung Medical University Hospital in Taiwan, and written informed consent was obtained prior to the data collection.

### Study instruments

The working group consisted of five authors with academic positions and teaching experiences in medical education and two faculties with expertise in medical education and questionnaire design. The first study instrument, namely, the Clinical Learning Environmental Questionnaire (CLENQ) questionnaire, was constructed through a focus group discussion process. The teaching dimension was mainly adopted from the “teaching subscale” of the PHEEM instrument [4]. We hypothesise that the other dimensions, such as clinical workloads, working and social interactions with patients and medical teams, and organisational culture, may differ between Taiwan and Western or other countries. Thus, new items of these dimensions of clinical learning environment were constructed in alignment with local medical training settings and the socio-cultural context. For example, the reference background of the items relating to workload was stemmed from the workload survey from the Taiwan Ministry of Labor (http://wecare.mol.gov.tw/lcs_web/contentlist_cN_p01.aspx?ProgId=10601001&SNO=2901) and job stress survey among first postgraduate year residents [30]. Specifically, “Item 3: When I am on call I am unable to get enough sleep” indicates heavy clinical workload under the current status of no working-hour regulation in Taiwan. Furthermore, “Item 24: I feel stressed when interacting with visiting staff” and “Item 8: I am afraid of causing a lawsuit” reflects the great tension of the power relationship between medical trainees and supervisors as well as the distinct social expectations of patients or family on medical professions in Taiwan.

The initial 50-item draft version of CLENQ was constructed under the consensus of the working group. Pilot validation of the questionnaire was conducted by ten medical trainees to make the final version of a 39-item CLENQ and one global scale item under the criteria of avoiding duplication and ambiguous expression. Each item of CLENQ was on a 5-point scale ranging from 1 to 5 (strongly disagree to strongly agree), yielding total scores from 40 to 200. Reverse scores were counted for negative statement items. The higher scores indicated more positive perceptions of clinical learning environment.

The second study instrument was a five-item Brief Symptoms Rating Scale (BSRS-5) for measuring the mental distress status of participants. BSRS has been used as a screening instrument for measuring the five psychological symptoms and behaviours [31, 33]. These include: (1) anxiety (Do you feel tense or high-strung in the past week?), (2) depression (Do you feel depressed or in a low mood in the past week?), (3) hostility (Do you feel easily annoyed or irritated in the past week?), (4) interpersonal sensitivity (Do you feel inferior to others in the past week?) and (5) insomnia (Do you have trouble falling asleep in the past week?). The BSRS-5 was administered using 5-point severity scales (0, not at all; 1, mild; 2, moderate; 3, severe; 4, profound). Possible total scores on the BSRS-5 ranged from 0 to 20, with higher scores indicating more severe mental distress [31, 33].

### Data management/statistical analyses

To validate the CLENQ, exploratory factor analysis and Cronbach α were conducted to investigate construct validity and internal consistency. Exploratory factor analysis was carried out using principle component analysis (PCA) followed by varimax rotation. Factor numbers were determined with the Scree Test, in which the eigenvalues of all the factors were examined after each factor was extracted until a major drop or discontinuity was observed, after which time the remaining factors were retained. Items with a loading score of >0.3 were entered into a factor. Then, based on the factor analysis results, these items were automatically loaded into the same factor. However, if the factor loading was >0.3 for more than two items, the author would judiciously define the appropriate factor for the items based on similarity of the statement meanings of this item with other items with higher factor loadings.

Analysis of variance tests (ANOVA) or chi square analyses were used to compare demographic data across groups. Correlations were calculated between total scores or subscale scores of factors of CLENQ and BSRS-5 total scores by controlling covariates.

## Results

In total, 189 of 450 medical trainees completed the questionnaires with a response rate of 42%. The demographic data of participants and departments of training are listed in Table 1. Mean age among three groups of interns, PGY1, and senior residents (Rs) were significantly different (F = 31.1, *p* < 0.001). Gender distribution did not differ among the three groups (X^2^ = 1.55; *p* = 0.46). The distribution of departments differed across three groups (X^2^ = 15.0, *p* = 0.005). As the distribution of department and mean of age differed across groups, we treated them as covariates when comparing scores BSRS-5 and CLENQ across the three groups and investigating the correlations between BSRS-5 and CLENQ by groups.

**Table 1:** **Demographic data and distribution of departments of clinical training across three levels of medical trainees**

	Intern	PGY1	Rs	***p***value*
N (%)	62 (32.8%)	52 (27.5%)	75 (38.7%)	
Mean age (SD)	26.2 (2.6)	27.8 (2.9)	30.0 (2.9)	<0.001
Gender				.46
Male	44 (71.0%)	40 (76.9%)	60 (80.0%)	
Female	18 (29.0%)	12 (23.1%)	15 (20.0%)	
Departments				0.005
Medicine	26 (41.9%)	16 (30.8%)	28 (37.3%)	
Surgery	21 (33.9%)	6 (11.5%)	17 (22.7%)	
Others	15 (24.2%)	30 (57.7%)	30 (40.0%)	

*PGY1* postgraduate year one residents, *Rs* senior residents.

*p for chi-square test.

Using Scree plot from the exploratory analysis, the loadings >0.3 yielded five factors as shown in Table 2. These five factors accounted for 46.5% of the total variance; each factor (I to V) was responsible for 19.0%, 11.2%, 6.9%, 5.4%, and 4.0% of total variance, respectively. According to the meanings of the items making up each factor, these five factors were labelled as: I: Teaching (13 items), II: Workload (7 items), III: Relationship pressure (9 items), IV: Organisational support (4 items) and V: Mutual trust (6 items). In total, 22 items were negative statements among factors of workload, relationship, and trust. One final item of the global scale did not belong to one of the five factors. Factor I (teaching) contained items relevant to roles and teaching performance of clinical teachers, overall atmosphere of the medical context educational practice in supervision, assessment, and feedback. Factor II (workload) contained items of job stress relating to clinical workload and adapting to the workplace, as well as coping with the job stress. Factor III (relationship pressure) contained items regarding social relationship pressure with patients and medical teams. Factor IV (organisational support) contained items involving the preparedness of learners’ pre-existing medical knowledge and skills as well as support received from colleagues and the organisation. Factor V (mutual trust) contained items relating to perceptions of inadequate self-confidence and mutual trust from patients and medical teams.

**Table 2:** **Factor Analysis of Clinical Learning Environment Questionnaire (CLENQ) across three levels of medical trainees**

	I	II	III	IV	V
	Teaching	Workload	Relationship pressure	Support	Trust
19. My clinical teachers are enthusiastic about teaching	**0.78**	0.07	−0.17	0.14	−0.02
15. All of my clinical teachers have good teaching skills	**0.74**	0.14	−0.18	0.03	0.03
21. My clinical teachers effectively utilise clinical cases when teaching me	**0.72**	0.12	−0.03	0.16	−0.13
28. My clinical teachers set up clear learning objectives for me	**0.71**	−0.15	−0.10	−0.02	0.18
33. Senior residents take the initiative to care about my clinical work	**0.69**	−0.07	0.26	0.07	−0.20
37. I feel that my clinical teachers are approachable	**0.69**	−0.07	−0.21	−0.04	−0.20
34. I regularly receive feedback from senior medical staff that helps me review and improve my clinical practices	**0.68**	−0.21	0.01	0.22	−0.01
38. My clinical teachers encourage me to learn independently to improve my clinical skills	**0.67**	−0.14	−0.05	−0.01	−0.16
18. During my clinical learning hours, I have good supervision and protection	**0.65**	−0.20	−0.02	0.28	0.18
14. Visiting staff take the initiative to care about my clinical work	**0.58**	0.09	−0.21	0.35	0.09
32. There are appropriate mechanisms in place to assess if my training plans meet my needs	**0.45**	−0.16	0.12	0.28	0.23
23. I like the current work environment and atmosphere	**0.44**	−0.35	0.09	0.38	−0.03
35. I have enough opportunity to learn the knowledge and skills I need to practice medicine	**0.41**	−0.29	0.21	0.23	−0.26
1. My workload is more than I can handle	−0.15	**0.79**	−0.06	0.06	0.15
2. My job takes a lot of my energy and physical strength	−0.09	**0.75**	−0.03	0.09	0.12
16. I have a heavy workload when I am on call	−0.01	**0.71**	0.17	−0.15	−0.05
3. When I am on call I am unable to get enough sleep	−0.18	**0.61**	0.21	0.16	−0.18
4. I do not receive appropriate compensation from my job	−0.19	**0.54**	0.24	−0.18	0.01
27. I am stressed when dealing with patients who present with problems outside my specialty when I am on duty	0.09	**0.48**	0.24	−0.03	0.13
5. 5. I am not familiar with the equipment in this hospital	0.12	**0.46**	0.13	−0.37	0.32
11. When patients or their family know that I am a new physician, they do not trust the medical treatment I provide	−0.01	0.08	**0.62**	−0.19	0.14
9. I feel stressed when communicating with patients or their family members	0.01	0.08	**0.58**	−0.33	0.25
10. I have been asked to treat patients based on cost, not on quality of care	−0.22	0.21	**0.58**	0.11	−0.22
24. I feel stressed when interacting with visiting staff	−0.19	0.03	**0.56**	−0.04	0.36
31. I did not learn enough about medical ethics to help me with my clinical decisions	−0.13	0.01	**0.56**	−0.08	0.18
8. 8. I am afraid of causing a lawsuit	0.01	0.23	**0.55**	0.10	−0.14
26. Other non-physician medical staff members do not trust my clinical decisions because I am a new physician	−0.16	0.05	**0.54**	−0.34	0.17
20. My colleagues and I are competitive	0.01	0.08	**0.47**	0.04	0.21
30. A patient’s or their family’s socioeconomic status affects my clinical decisions about their care	0.07	0.08	**0.45**	0.09	0.18
22. The knowledge and skills I have learned help me in dealing with clinical emergencies	0.24	−0.04	0.06	**0.68**	−0.01
17. Nursing staff members help me with my clinical work	0.33	−0.06	−0.05	**0.59**	0.19
25. I get along with non-physician medical staff members	0.23	−0.04	−0.21	**0.56**	0.01
7. The knowledge and skills I have learned help me to finish my work	0.22	0.15	0.06	**0.35**	−0.19
36. I feel stressed when interacting with senior residents	−0.14	−0.10	0.21	0.02	**0.69**
39. Visiting staff don’t trust my clinical decisions because I am a new physician	−0.04	−0.04	−0.29	0.10	**0.60**
13. I feel anxious and helpless when my patient is dying	0.19	0.03	0.10	−0.47	**0.50**
6. I do not have enough confidence in my clinical skills	0.04	0.32	0.22	−0.31	**0.42**
29. I am unfamiliar with the medical system that helps me take care of patients	0.01	0.38	0.26	−0.29	**0.34**
12. I feel lonely and helpless when I am on call	−0.03	0.06	0.07	−0.01	**0.32**
Cronbach’s	0.89	0.79	0.76	0.62	0.71
Eigen value	7.42	4.37	2.68	2.12	1.57
Explained variances	19.04%	11.20%	6.86%	5.42%	4.01%

Bold numbers denote the loadings of item in each factor.

Based on the items of CLENQ making up the newly extracted factors, the analyses of internal consistency revealed Cronbach’s α values of 0.89, 0.79, 0.76, 0.62, and 0.71 for factors I-V, respectively. The overall Cronbach’s α for the total CLENQ was 0.88. Interfactor correlation coefficients ranged from 0.17 (factor I and factor V) to 0.54 (factor I and factor IV).

We summed the subscale scores of each factor. The higher scores of factors from CLENQ indicated more positive perceptions of the clinical learning environment and, accordingly, better perceptions of teaching, less workload stress, less social relationship pressure, better organisational support, and better trust.

Furthermore, we compared the mental distress as measured by BSRS-5 and perceptions of clinical learning environment across the three groups using ANOVA tests (Table 3). The intern group showed higher scores of BSRS-5, however, the difference did not reach statistical significance after controlling for covariates. By controlling for covariates of department and age, total scores of CLENQ and subscales of “perceptions of teaching” and “perceptions of organisational support” were lower in the intern group than in PGY1 and Rs groups. Furthermore, the subscale of “perceptions of social relation pressure” was lower in intern and Rs groups. In contrast, the subscale of “perceptions of trust” was lower in groups of interns and PGY1.

**Table 3:** **Mental well-being and perceptions of clinical learning environment across three levels of medical trainees**

	Intern N = 62	PGY1 N = 52	Rs N = 75	Statistics	Post-hoc comparisons
BSRS-5	8.3 (4.5)	6.4 (4.2)	6.7 (4.5)	F = 0.53	
				*p* = 0.59	
CLENQ					
Total	118.8 (13.9)	128.7 (14.3)	127.4 (12.5)	F = 5.50	PGY1, Rs > Int
				*p* = 0.005	
Teaching	44.1 (7.5)	49.2 (5.2)	47.5 (5.9)	F = 7.22	PGY1, Rs > Int
				*p* = 0.001	
Workload	17.1 (4.5)	18.5 (4.6)	18.7 (4.0)	F = 0.99	
				*p* = 0.38	
Relationship pressure	24.8 (4.8)	27.0 (5.0)	24.8 (4.6)	F = 3.26	PGY1 > intern, Rs
				*p* = 0.041	
Support	15.0 (2.2)	15.8 (1.5)	15.9 (1.5)	F = 4.66	PGY1, Rs > Int
				*p* = 0.011	
Trust	17.9 (3.3)	18.2 (3.7)	20.4 (3.3)	F = 6.65	Rs > Int, PGY1
				*p* = 0.002	

Presented with mean (SD).

*Int* intern, *PGY1* postgraduate year one residents, *Rs* senior residents.

*BSRS-5* five-item Brief Symptoms Rating Scales.

The correlations between BSRS-5 and CLENQ and the total score or subtotal scores of each factor are shown in Table 4. Total scores of BSRS-5 were negatively correlated with the total score of CLENQ regardless of groups. Further analysis revealed that BSRS-5 was negatively correlated with factor I “perceptions of teaching” in the PGY1 group alone and with factor II “perceptions of workload” in all three groups. Moreover, BSRS-5 was negatively correlated with factor III “perceptions of social relationship pressure” and with factor V “perceptions of trust” in both PGY1 and Rs groups. However, there was no significant correlation between BSRS-5 and factor IV “perceptions of organisational support” across the three groups.

**Table 4:** **Correlations between mental well-being and perceptions of clinical learning environment across three levels of medical trainees**

		BSRS-5 total	
	Intern	PGY1	Rs
CLENQ			
Total	r = −0.29, *p* = 0.025	r = −0.62, *p* <0.001	r = −0.44, *p* <0.001
Teaching	r = −0.21, *p* =0.11	r = −0.32, *p* =0.022	r = −0.18, *p* =0.12
Workload	r = −0.34, *p* =0.007	r = −0.50, *p* <0.001	r = −0.38, *p* =0.001
Relationship pressure	r = −0.05, *p* =0.72	r = −0.53, *p* <0.001	r = −0.27, *p* =0.022
Support	r = −0.06, *p* =0.62	r = −0.07, *p* =0.60	r = −0.18, *p* =0.12
Trust	r = −0.19, *p* =0.14	r = −0.54, *p* <0.001	r = −0.44, *p* <0.001

*PGY1* postgraduate year one residents, *Rs* senior residents.

*BSRS-5* five-item Brief Symptoms Rating Scales.

*CLENQ* Clinical Learning Environment Questionnaire.

## Discussion

This study has demonstrated that the CLENQ instrument could be categorised into five subscales ranging from factor I to V, indicating perceptions of teaching, workload, social relationship pressure, organisational support, and mutual trust, respectively. The high Cronbach’s α value for total scores and all subscales indicated good internal consistency. This suggests that CLENQ has good reliability and construct validity as an instrument for measuring the clinical learning environment for our medical trainees. The strongest factor was perceptions of teaching. This finding was consistent with the original PHEEM report [4] and the international multi-centre study [6]. It indicates that the teaching dimension is still the strongest factor for medical trainees in assessing clinical learning environment. The top 4 items of teaching factor focussed on the clinical teachers’ enthusiasm, teaching skills, case-based teaching methods, and clear learning objectives. The second strongest factor was perceptions of workload. Clinical work overload has been reported as a critical factor relating to burnout in the surveys from Taiwan [30] and most other centres [26, 27, 34]. Workload was attributed to heavy job demands, long duty hours, frequent calling and deprivation of sleep, which are the priority issues that need to be solved.

The third factor was perceptions of social relationship pressure with patients and medical teams. There are two explanations for the findings on relationship pressure factor. First, the growing medical legal disputes may lead to tense relationships between physicians and patients. Based on a statistics report of Department of Health in Taiwan, medical lawsuits under trial at court rose from 127/year in 2000 to 545/year in 2010 [35]. Second, it might be due to our distinct socio-cultural factors. Hofstede’s international study on culture and organisations has demonstrated that the power distance index in Taiwan ranks at 43-44/76 countries and is higher than in Western countries [36]. The organisational culture of high power distance may exert the emotional distress for our medical trainees when interacting with supervisors or senior staff.

The fourth factor, perceptions of organisational support, has demonstrated that organisational support is needed and helpful when medical trainees’ pre-existing knowledge and skills are inadequate for clinical practice [23, 25, 26]. Provision of preparatory courses and actual experience improved medical trainee’s performance in transitions [37].

The last factor V, perceptions of trust, may reflect the medical trainees’ sense of insecurity and immature self-identity [38], as well as difficulties in playing their new role within the medical team [25]. According to Hofstede [36], the cultural factor of uncertainty avoidance refers to the extent to which the members of the culture feel threatened by ambiguous or unknown situations. Taiwan’s uncertainty avoidance index, ranking at 39/76 countries, is higher than that of most Western countries. This socio-cultural factor may lead to high levels of anxiety and job stress, feeling of insecurity and lack of team trust in our medical trainees.

Younger medical trainees (interns) seem to report the lower scores in total and most subscales of CLENQ in comparison to advanced trainees (PGY1 and senior residents), especially in subscales of teaching and support. Several reasons may account for the unfavourable perceptions of clinical learning environment by interns. First, interns usually receive training with frequent rotation at different departments of multiple medical disciplines. Teaching programs may become fragmented [27]. Second, a lack of preparation and induction courses may also impede medical performance during the period of transition [37, 39].

Although five factors have been classified based on exploratory factor analysis in this CLENQ, there are several overlapping items that could be categorised into more than one factor. For instance, item 36 and 39 in the factor “trust” are also related with factor “relationship pressure”. Similarly, item 11 in the factor “relationship pressure” also touches upon the factor “trust”. Notably, a recent report from Sweden and Netherlands also demonstrated the overarching dimensions of experiential learning and social participation in a new undergraduate clinical education environment measure [40]. Previous studies also argued whether PHEEM was a single or multi-dimensional instrument [6, 7]. Moreover, “relationship pressure factor” could be divided into either “internal self” sub-factor (item 10 and 31) or “external interrelations” sub-factor (item 8, 9, 11, 24, and 26). Further studies with larger samples are needed to clarify these points.

As for mental distress status, although BSRS-5 scores were similar across three levels of medical trainees, there were negative correlations between BSRS-5 and both total CLENQ scores and workload subscale for all three groups. This suggests that the quality of overall clinical learning environment, especially workload factor, may play a key role in affecting the trainee’s mental distress status. Moreover, BSRS-5 scores were negatively correlated with perceptions of relationship pressure and trust in PGY1 and senior resident groups rather than in the interns’ group. Since the more senior trainees need to take care of the more complicated patients, they may encounter complex social relationships and difficult situations of trust with patients and the medical teams, which may explain the negative correlation just mentioned. In Taiwan, interns do not have medical licence yet and they are only allowed to carry out less invasive clinical tasks under strict supervision. It is worthy to investigate whether BSRS-5 affects the quality of life in medical trainees [9].

Our study may enrich the literature by providing the socio-cultural relevance and medical contextual significance in designing and measuring the clinical educational environment. First, it has reemphasised the importance of perceptions of teaching, in agreement with PHEEM study [4]. Second, our study has identified three crucial factors of clinical learning environment: workload, social relationship pressure and mutual trust. Increasing workload may derive from the insufficient physician manpower and long duty hours. It urges to call for governmental regulation of the working duty hours and also to drive the public paying more attention to the welfare of medical trainees. Third, the findings from both social relationship pressure and mutual trust factors are in accordance with the Hofstede’s conceptual framework of socio-cultural factors in organisations and nations [36, 41]. Therefore, our approach of incorporating local socio-cultural factors and medical training settings into CLENQ design is not only greatly relevant to Taiwan but also crucially significant and applicable to other countries.

However, the results of this study must be interpreted with caution due to several limitations. First, study participants of medical trainees from a single teaching hospital were limited in number and the response rates were relatively low. The explanation for this is that our medical trainees need to take clinical rotations frequently in different departments and three affiliated hospitals. Second, the cross-sectional design made it difficult to determine the causal relationship between CLE and mental distress variables. Third, the construct of CLENQ instrument was not validated or tested in other socio-cultural contexts. These weaknesses may limit the generalisation of the findings and conclusions of this study.

## Conclusions

Our study using CLENQ has identified five major factors of clinical learning environment that are closely linked with our local socio-cultural factors and medical training settings. Medical trainee’s mental distress status was negatively correlated with the quality of CLENQ. These findings have socio-cultural relevance and medical contextual significance and might be applicable to other countries. It warrants further study to investigate the impact of clinical learning environment improvement on the medical trainee’s mental distress and performance.
